# Supplementary material for: The T Cell Immunoscore as a Reference for Biomarker Development Utilizing Real-World Data from Patients with Advanced Malignancies Treated with Immune Checkpoint Inhibitors
Source: Cancers (Basel). 2023 Oct 10;15(20):4913. doi: 10.3390/cancers15204913 (PMC10605389; doi:10.3390/cancers15204913)
Supplement: Supplementary file 1 [file cancers-15-04913-s001.zip › cancers-2601811-supplementary.pdf]

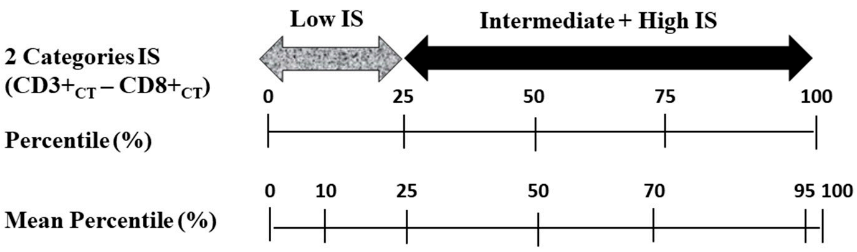

Figure S1. Immunoscore calculation using two categories of "low" and "intermediate-high" using imputed densities of $CD3+_{CT}$ and $CD8+_{CT}$

Abbreviation: CD; cluster differentiation, CT; center of the tumor, IS; immunoscore.

Adapted from Galon *et al*. [1].
